# Intrinsic Folding Properties of the HLA-B27 Heavy Chain Revealed by Single Chain Trimer Versions of Peptide-Loaded Class I Major Histocompatibility Complex Molecules

**DOI:** 10.3389/fimmu.2022.902135

**Published:** 2022-07-25

**Authors:** Izabela Lenart, Linh-Huyen Truong, Dinh Dung Nguyen, Olga Rasiukienė, Edward Tsao, Jonathan Armstrong, Pankaj Kumar, Kirsty McHugh, Branca I. Pereira, Balraj S. Maan, Malgorzata A. Garstka, Paul Bowness, Neil Blake, Simon J. Powis, Keith Gould, Darren Nesbeth, Antony N. Antoniou

**Affiliations:** ^1^ Division of Infection and Immunity/Centre of Rheumatology, University College London, London, United Kingdom; ^2^ Centre of Rheumatology, University College London, London, United Kingdom; ^3^ Clinical Trials and Regulatory Affairs, Science Pharma, Warsaw, Poland; ^4^ Department of Applied Sciences, Faculty of Health and Life Sciences, Northumbria University, Newcastle upon Tyne, United Kingdom; ^5^ University of Oxford, Botnar Research Centre, Nuffield Department of Orthopaedics, Rheumatology and Musculoskeletal Sciences, Oxford, United Kingdom; ^6^ Medical Genetics Department, Medical Genetics centre, Vinmec Research Institute of Stem Cell and Gene Technology, Hanoi, Vietnam; ^7^ School of Medicine and Biological Sciences Research Complex, University of St. Andrews, Scotland, United Kingdom; ^8^ The Nuffield Department of Orthopaedics Rheumatology and Musculoskeletal Science, Oxford University, Oxford, United Kingdom; ^9^ Jenner Institute, University of Oxford, Oxford, United Kingdom; ^10^ Research and Development Department, Chelsea and Westminster Hospital National Health Service (NHS) Foundation Trust, London, United Kingdom; ^11^ School of Medical Education, The Faculty of Medical Sciences, Newcastle University, Newcastle Upon Tyne, United Kingdom; ^12^ Core Research Laboratory, Department of Endocrinology, National & Local Joint Engineering Research Center of Biodiagnosis and Biotherapy, Second Affiliated Hospital, School of Medicine, Xi’an Jiaotong University, Xi’an, China; ^13^ Institute of Infection and Global Health, University of Liverpool, Liverpool, United Kingdom; ^14^ Wright-Fleming Institute, Imperial College London, London, United Kingdom; ^15^ The Advanced Centre for Biochemical Engineering, University College London, London, United Kingdom

**Keywords:** HLA-B27, ankylosying spondylitis, MHC class I misfolding, single chain trimers, F pocket, HLA-B27 alleles

## Abstract

Peptide-loaded Major Histocompatibility Complex (pMHC) class I molecules can be expressed in a single chain trimeric (SCT) format, composed of a specific peptide fused to the light chain beta-2 microglobulin (β2m) and MHC class I heavy chain (HC) by flexible linker peptides. pMHC SCTs have been used as effective molecular tools to investigate cellular immunity and represent a promising vaccine platform technology, due to their intracellular folding and assembly which is apparently independent of host cell folding pathways and chaperones. However, certain MHC class I HC molecules, such as the Human Leukocyte Antigen B27 (HLA-B27) allele, present a challenge due to their tendency to form HC aggregates. We constructed a series of single chain trimeric molecules to determine the behaviour of the HLA-B27 HC in a scenario that usually allows for efficient MHC class I molecule folding. When stably expressed, a pMHC SCT incorporating HLA-B27 HC formed chaperone-bound homodimers within the endoplasmic reticulum (ER). A series of HLA-B27 SCT substitution mutations revealed that the F pocket and antigen binding groove regions of the HLA-B27 HC defined the folding and dimerisation of the single chain complex, independently of the peptide sequence. Furthermore, pMHC SCTs can demonstrate variability in their association with the intracellular antigen processing machinery.

## Introduction

Major Histocompatibility Complex (MHC) class I molecules are comprised of a tripartite complex of heavy chain (HC), beta-2 microglobulin (β2m) and peptide. MHC class I-peptide complexes assemble within the lumen of the endoplasmic reticulum (ER). The assembly of MHC class I-peptide complexes involves a series of interactions with lectins, soluble chaperones and oxidoreductases. Partially folded MHC class I complexes are formed following interactions with calnexin (CNX), calreticulin (CRT) and Immunoglobulin Binding Protein (BiP) (1–3), along with the activity of the protein disulfide isomerase (PDI) family of oxidoreductases, ERp57 and PDI (4–9). These partially folded MHC class I complexes form the peptide loading complex (PLC), where they are tethered to the transporter associated with antigen presentation (TAP) heterodimer *via* the MHC class I specific accessory protein tapasin, which is disulfide bonded to ERp57 (10–12). Within the PLC, partially folded MHC class I-β2m complexes are associated with CRT and PDI (4, 13–15). The principal function of the PLC is the acquisition of optimal peptide *via* the catalytic activity of tapasin (15). Furthermore there exist a further MHC class I molecule specific peptide exchange catalyst, the transporter associated with antigen processing binding protein-related (TAPBPR) protein, which operates outside the PLC and in conjunction with the folding sensor UDP-glucose:glycoprotein glucosyltransferase 1 (UGT1) (16).

Single chain trimeric (SCT) molecules are composed of MHC class I heavy chains (HC) physically linked to each member of the tripartite complex through a series of linkers. The SCT format, which allows for efficient folding is comprised of the appropriate peptide at the amino (N)-terminus followed by a flexible linker connecting the carboxy (C)-terminus of the peptide to the N-terminus of β2m, followed by a second flexible linker and the MHC class I HC (17–20). These molecules have been demonstrated to fold efficiently and generate strong T cell responses (18, 21, 22). Due to the physical linkage of all three components, it has been proposed that SCTs could bypass intracellular antigen processing pathways (23), which was demonstrated by their efficient expression in TAP deficient cell lines (24, 25). The physical linkage of peptide would obviate the requirement for peptide processing, optimization and exchange reactions. SCT molecules have been widely used as biological probes for lymphocyte development, activation and to enumerate disease related T cells (18, 26, 27). Importantly, SCT molecules have been engineered for clinical applications. The SCT format of MHC class I-peptide complexes act as efficient vaccine vehicles, overcoming the limitations of antigen-based DNA vaccines. SCTs can stimulate anti-tumour specific responses to weak MHC class I binding tumour specific peptides and potentially overcome MHC class I down modulation mechanisms such as those employed by viruses and some tumours (17, 23, 28, 29). In addition, certain MHC SCT molecules such as Human Leukocyte Antigen (HLA)-E have been employed to circumvent rejection of cellular immunotherapeutic modalities (30, 31).

HLA-B27 is an MHC class I molecule strongly associated with the group of inflammatory arthritic diseases known as the spondyloarthropathies (SpAs) (32, 33). Misfolding of the HLA-B27 HC has been proposed to play a significant role in SpA disease (34–40). The HLA-B27 monomeric HC can misfold and exhibt prolonged maturation times, as determined by acquisition of endoglycosidase H (endo H) resistence (3, 20, 40). HLA-B27 HCs can misfold into unusual conformations and exhibit enhanced ER associated degradation (20, 40). Additionally, HLA-B27 can form HC-dimeric conformations which have been proposed to contribute to SpA pathogenesis (34–36).

MHC class I HCs are comprised of three distinct extracellular domains, the α1, α2 and α3 domains, a transmembrane and cytosolic domains. The extracellular domain has two conserved disulfide bonds, one within the α2 domain between cysteines (C)101-C164 and a second within the α3 domain between C203-C259. MHC class I HCs can express unpaired cysteines such as HLA-G at position(p) 42 (41) and HLA-B alleles at p67, p308 and p325, with p308 thought to interact with tapasin (42).

HLA-B27 expresses a unique combination of three unpaired cysteine residues at p67, 308 and 325. C67, which contributes to the B pocket of the antigen binding groove, has been demonstrated to participate in the formation of different HLA-B27 dimeric complexes. HLA-B27 HC-dimeric populations are heterogenous and can exist as ‘unfolded’ or ‘folded’ forms and in different redox states (39, 43, 44). C67 can participate in recombinant HLA-B27 HC dimerisation, whilst *in vivo* it can contribute to both ‘unfolded’ or ‘folded’ dimeric complexes (39, 45). Furthermore, the role of C67 dimerisation was proposed to partly depend on an adjacent lysine at p70, resulting in cysteine reactivity (3). C67 and C325 have been demonstrated to mediate HC-dimerisation at the cell surface and within exosomes respectively (46, 47), whilst the conserved structural C164 participates in dimerisation within the ER (3, 44). Aberrant dimeric conformations also depend on non-cysteine residues expressed within the antigen binding groove of HLA-B27 (48). The HLA-B*27:06 subtype, which is not associated with the SpAs, expresses aspartic acid (D) and tyrosine (Y) residues at p114 and p116 respectively within the F pocket of the antigen binding groove. In a peptide-limiting system, HLA-B27 molecules expressing D114Y116 exhibited reduced dimerisation and enhanced maturation kinetics (48). Furthermore, B pocket sequences other than C67 were also shown to influence HLA-B27 HC-dimers (39). These observations suggested the folding status of HLA-B27 was either related to inherent qualities within the heavy chain itself or affected by a distinct subset of peptides.

Here we have generated HLA-B27 SCT molecules to address the questions of whether the HLA-B27 HC possesses inherent misfolding properties and the extent to which F pocket amino acid residues influence folding, independent of the peptide sequence. In addition, by employing various HLA-B27.SCT molecules we were able to examine the extent MHC class I SCT molecules are independent of the antigen processing machinery.

## Materials and Methods

### Cells Lines and Antibodies

HLA-B27 SCT molecules were generated as described previously (20, 49). The SCT molecule comprised the human β_2_m amino-terminal hydrophobic signal sequence, the peptide sequence SRYWAIRTR (residues 383–391 of influenza virus nucleoprotein NP), a GGGGGG(SGG)_3_ linker, human β_2_m sequence, a second linker (GGGGS)_3_ and HLA-B*27:05 class I HC sequence, which was C-terminally V5 tagged. For the HLA-B*35:01 SCT, the EBNA1 Human Papilloma Virus peptide sequence HPVGEADYFEY was employed.

The Invitrogen Flp-In™ system was used to generate isogenic HeLa lines stably expressing two copies of HLA class I constructs driven by the human cytomegalovirus (HCMV) promoter (49). Separate cell lines were generated to express the HLA-B*27:05 or –B*35:01 HCs and an empty vector (referred to subsequently as Empty (E)84). HLA-B27 SCT molecules expressing C67S, C308S, C325S and H114DD116Y (referred to as F pocket) were generated using Quikchange PCR site directed mutagenesis. Sequences were verified using the Dundee University sequencing service. All lines were genetically identical and expressed only two copies of the above constructs. Cells were maintained in DMEM, supplemented with 10% FBS (Globepharm), penicillin/streptomycin (D10 media), hygromycin (150μg/ml) and maintained in a humidified 5% CO_2_ 37°C incubator.

Monoclonal antibody ME1 (50), recognising folded HLA-B molecules was kindly provided by Dr. J. Taurog and W6/32 (51) recognising folded HLA-A, B and C molecules was kindly provided by Prof. T. Elliott. Monoclonal antibody HC10 recognises unfolded HLA-B and –C molecules. Anti-V5 antibody was from Serotec, LAMP1-PE antibody (CD107a) (clone H4A3) was purchased from BD-Pharmingen (555801) and CD8-APC antibody (clone RPA-T8) was purchased from BD-Pharmingen (561953). Goat anti-mouse horseradish peroxidase (HRP) and conjugated secondary antibody were purchased from DAKO. Anti-tapasin PASTA antibody was a kind gift from Prof. P. Cresswell and anti-TAP1 monoclonal antibody were obtained from Stressgen.

### Flow Cytometry

Cells were trypsinized, washed and fixed for 10 mins with 1.8% paraformaldehyde pH7.4. Cells were acquired on an LSR Fortessa or BD Canto II (BD Biosciences), using FACSDiva software and analysed using FlowJo 8.7.3.

### Cytotoxic T Lymphocyte (CTL) Assays

HLA-B27 heterotrimeric tetramer complexes generated as previously described were used to stimulate and expand viral peptide-specific HLA-restricted CD8^+^ cytotoxic T cell lines from HLA-B27^+^ peripheral blood monocytic cells (PBMC). T cell activation was assessed using CD107a and CD8 expression as previously described (49). Heparinized venous blood was obtained from patients with AS (modified New York criteria), with ethical permission (COREC 06/Q1606/139 and Oxfordshire Research Ethics Committee B 07/Q1605/35) and upon informed consent.

### Peptide Mass Spectrometry Analysis

One confluent 175cm^2^ flask of each cell lines was lysed in1% NP40 buffer (5ml) and immunoprecipitated with sepharose beads (300μl) coupled to W6/32. Beads were washed extensively with NaCl (150mM) Tris-HCl (10mM, pH7.6) to remove the detergent and resuspended in 1% Trifluoroacetic acid (TFA). The eluted HLA class I and peptides were isolated using C18 microtips (Thermofisher Scientific, UK) following manufacturer instructions and eluted in 30% acetonitrile, 0.15% TFA. Peptides were then analyzed on an AB Sciex TripleTOF 5600 system mass spectrometer (Sciex, Framingham, MA) coupled to an Eksigent nano LC AS-2/2Dplus system. The samples were resuspended in loading buffer (2% acetonitrile and 0.05% trifluoroacetic acid) and bound to an Aclaim pepmap 100mm x 2-cm trap (Thermo Fisher Scientific) and washed for 10 mins to waste after which the trap was turned in-line with the analytical column (Aclaim pepmap RSLC 75mm x 15 cm). The analytical solvent system consisted of buffer A (2% acetonitrile and 0.1% formic acid in water) and buffer B (2% water with 0.1% formic acid in acetonitrile) at a flow rate of 300 nl/min with the following gradient: linear 1–20% of buffer B over 90min, linear 20–40% of buffer B for 30 min, linear 40–99% of buffer B for 10 min, isocratic 99% of buffer B for 5 min, linear 99–1% of buffer B for 2.5 min and isocratic 1% solvent buffer B for 12.5 min. The mass spectrometer was operated in the DDA top 20 positive ion mode, with 120 and 80ms acquisition time for the MS1 (*m*/*z* 400–1250) and MS2 (*m*/*z* 95–1800) scans, respectively, and 15s dynamic exclusion. Rolling collision energy was used for fragmentation. Peak lists were generated within PeakView by using the “create mgf file” script. The MASCOT search engine with the following search parameters was used to identify peptides: no enzyme specificity, maximum of 4 miscleavages, oxidation as variable modification, peptide tolerance was set to 20ppm, and the MS-MS tolerance to 0.1 Da. Data were searched against the Swiss Prot database. Peptides identified in the Mascot files were assessed for their potential allele-binding specificity and affinity using NetMHC 4.0 for HLA alleles HLA-A*68, -B*15 and -B*27 (52).

### Immunoprecipitations

Pulse-chase, lysis, immunoprecipitations, and endoglycosidase H (endo H; Roche Applied Science) digestions were performed as previously described (3, 8, 53). For PLC immunoprecipitations, cells were pretreated with the alkylating agent N-ethylmalemide (NEM 20mM, pH7.4) (8, 54), lysed in 1% digitonin (WAKO), precleared and immunoprecipitated with either anti-TAP1, or –tapasin antibodies, resolved on non-reducing 8% SDS-PAGE gels and immunoblotted. For 2D gel analysis, 1% NP40 lysates or anti-tapasin immunoprecipitates were resolved according to charge in the first dimension and by Mw using non-reducing SDS-PAGE in the second dimension, followed by immunoblotting (55).

### Endoglycosidase H and PNGase F Digestions

1 x 10^6^ cells were harvested, incubated with NEM and then lysed in 1%NP40 (100μl) on ice for 30 mins and clarified by centrifugation. 10μl of lysates were incubated with 500u of either endoglycosidase H or PNGase F at 37°C for 1 hour, followed by resuspension in non-reducing sample buffer. Digests were then resolved by non-reducing or reducing SDS-PAGE, followed by immunoblotting for V5.

### Cell Surface Biotinylation

Confluent wells of a 6 well plate were washed with PBS and cell surface biotinylated by addition of EZ-Link Sulfo-NHS-biotin (1ml, 0.5 mg/ml, Thermo Fisher Scientific, UK) for 10 mins at room temperature. Cells were then lysed in 1% NP40 lysis buffer, immunoprecipitated with ME1, W6/32 and HC10 bound to Protein G-Sepharose beads (30μl), washed and analysed on non-reducing SDS-PAGE, transferred to nitrocellulose (BA45) and biotinylated proteins probed with Streptavidin-Dylight680 (Licor, UK) and scanned on a Licor Odyssey.

## Results

### Single Chain Trimer (SCT) Construct Design and Expression

**Figure 1:**
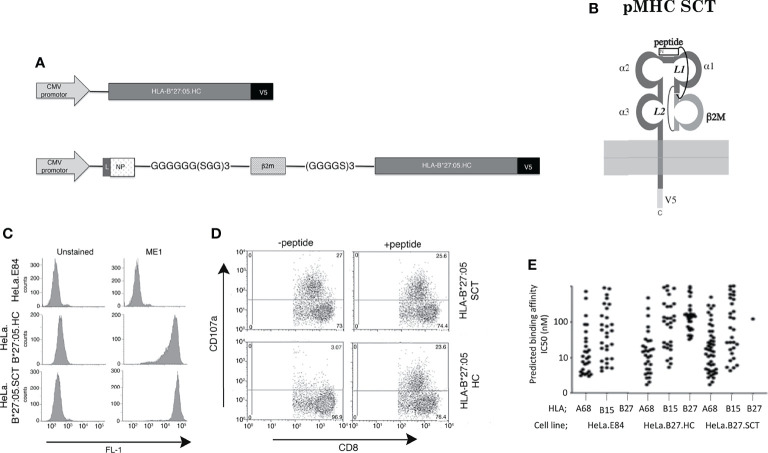
Construction and T cell recognition of HLA class I single chain trimeric (SCT) molecules; **(A)** Schematic outlining the HLA-B*27:05.heavy chain (HC) and HLA-B*27:05.SCT construct composed of huβ2m leader sequence (L)-SRYWAIRTR (NP) peptide-GGGGGG(SGG)_3_ linker-huβ2m-(GGGGS)_3_ linker-B27 heavy chain and V5 epitope tag. **(B)** Diagram of the HLA-B27.SCT. molecule composed of an N-terminal peptide linked to the N-terminus of β2m by linker 1 (L1). B2m is linked to the α1 domain of the MHC class I HC *via* linker 2 (L2) and the MHC class I HC was tagged (V5) at the C-terminus. **(C)** Cell surface expression of HLA-B*27:05 heavy chain and SCT formats expressed using the FLPIN system under the control of the HCMV promoter as determined by ME1 staining. **(D)** Influenza specific HLA-B*27:05 restricted CTL lines can efficiently be activated by HLA-B*27:05.SCT as well as HLA-B*27:05.HC lines pulsed with NP specific peptide. **(E)** Peptides were eluted and analysed by mass spectrometry and HLA class I binding affinity prediction from W6/32 immunoprecipitations of the cell lines. A significant number of HLA-B*27:05 binding peptides was detected only in the HLA-B*27:05.HC cell line, with only a single peptide detected in HLA.B*27:05.SCT and none in control HeLa.E84 line.

Isogenic HeLa cell lines expressing two copies of transgenes encoding HLA-B*27:05 class I HC or HLA-B*27:05 class I SCT under the control of the Human Cytomegalovirus (HCMV) promotor were generated as previously described (20, 49, 56) (**Figures 1A, B** **)**. The SRYWAIRTR peptide was employed as this exhibited high affinity binding to HLA-B*27:05 and exhibited similar binding affinities to the HLA-B*27:06 and 04 subtypes (57). An isogenic cell line expressing an empty vector was generated as a control (E84). Flow cytometric analysis with the conformationally specific ME1 antibody revealed that HLA-B*27:05.HC and SCT molecules were expressed efficiently at the plasma membrane of the respective cell lines (**Figure 1C**). CTL assays demonstrated that HLA-B*27:05.HC incubated with NP_383-391_ peptide acted as an efficient T cell target. The HLA-B*27:05.SCT activated CD8^+^ T cells in the presence and absence of exogenously added NP_383-391_ peptide (**Figure 1D**). To determine whether the associated peptide or other exogenous peptides bound the SCT, the monoclonal antibody W6/32 was used to immunoprecipitate MHC class I molecules (including HLA-B*27:05.SCT or -B*27:05.HC) from each cell line. Bound peptides were acid-eluted and analysed by mass spectrometry. The essentially complete absence of peptide signals in the data confirmed that HLA-B*27:05.SCT molecules almost exclusively associated with the covalently linked NP_383-391_ peptide (and therefore no free peptides were detected by mass spectrometry) whereas HLA-B*27:05.HC loaded a range of peptides (**Figure 1E**). The single ‘free’ peptide detected bound by HLA-B*27:05.SCT molecules, GRIGVITNR, was also detected in the pool of peptides eluted from B*27:05.HC molecules (**Supplementary Files S2** and **S3**). The complete list of peptides detected for all three HLA-I alleles in each cell line are presented in **Supplementary Files S1** **–** **S3**.

The HLA-B*27:05.SCT under a controlled transgene copy number integration system exhibits efficient cell surface expression which can act as a target for specific CTLs and binds predominantly a single peptide species. Therefore, our data with respect to the HLA-B*27:05.SCT are in line with reports of other previously generated pMHC class I SCTs (17, 21, 25, 28, 58–60).

### HLA-B*27:05.SCT Can Form HC-Dimers

**Figure 2:**
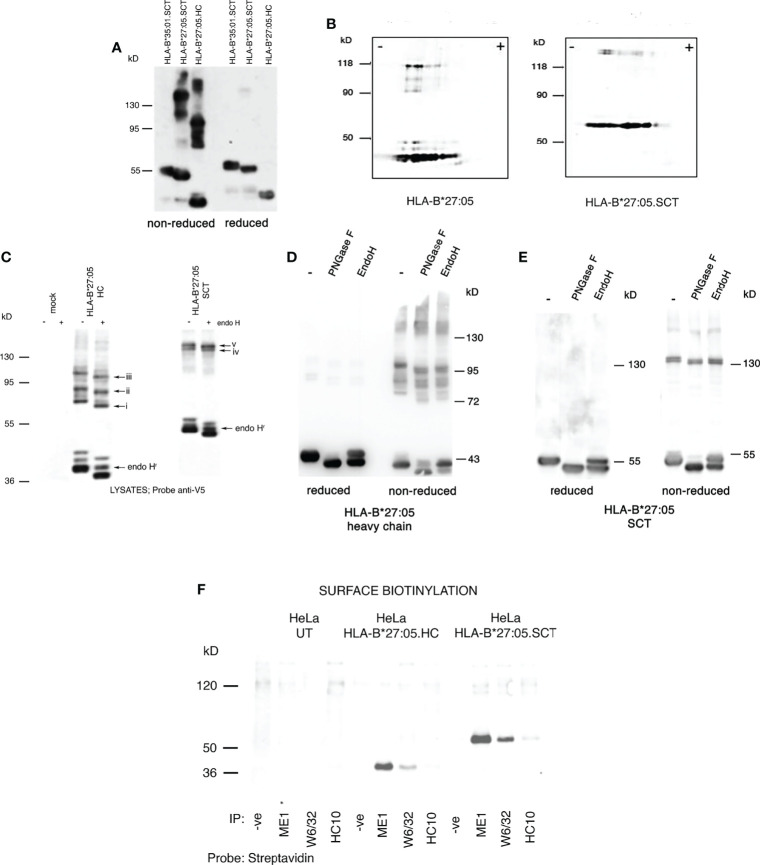
HLA-B*27:05.SCT form predominantly ER resident and HC dimeric structures; **(A)** Immunoblotting for MHC class I heavy chain with anti-V5 antibody of cell lysates from HLA-B*27:05 SCT, HLA-B*27:05 HC and HLA-B*35:01.SCT expressing cell lines, resolved by non-reducing SDS-PAGE, reveals that HLA-B*27:05 SCT can form dimeric complexes. **(B)** Immunoblotting for MHC class I heavy chain with anti-V5 antibody of non-reduced cell lysates from HLA-B*27:05 SCT and HLA-B*27:05 HC expressing cell lines, resolved by two-dimensional isoelectric focusing gel electrophoresis, reveals the HLA-B27 signal resolved as monomers and dimers with identical pI. **(C)** Immunoblotting for MHC class I heavy chain with anti-V5 antibody of non-reduced cell lysates from HLA-B*27:05 SCT and HLA-B*27:05 HC expressing cell lines, following endoH digestion and resolved by non-reducing SDS-PAGE reveals that HLA-B*27:05.SCT and HC dimers exhibited endo H sensitivity and were therefore predominantly ER resident. Arrows i-iii indicate shift in Mw due to N-glycan removal from each of the distinct HLA-B*27:05 dimer conformers. Arrows iv-v indicate HLA-B*27.SCT dimers were endo H sensitive. Endo H resistant conformers of HLA-B*27:05 HC and SCT are highlighted by arrow endoH^r^. **(D, E)** Immunoblotting for MHC class I heavy chain with anti-V5 antibody of lysates from HLA-B*27:05.HC and SCT expressing cell lines, following PNGase F and endo H digestion, resolved by non-reducing and reducing SDS-PAGE reveals a significant proportion of HLA-B*27:05.HC and SCT molecules remain in an immature state as determined by sugar modifications. Both HLA-B*27:05 HC and SCT high molecular weight complexes exhibit PNGase F and endo H sensitivity. **(F)** Immunoprecipitation with W6/32, ME1 and HC10 following cell surface biotinylation and resolution by non-reducing SDS-PAGE revealed no cell surface expression of HLA-B*27:05.SCT or HLA-B*27:05.HC dimers.

We wanted to determine the folding status of HLA-B*27:05.SCT under our experimental conditions. HLA-B*27:05.HC, HLA-B*27:05.SCT and HLA-B*35:01.SCT expressing HeLa cell lines were pre-treated with the alkylating agent NEM to trap disulfide bonds and prevent post lysis oxidation (8, 54), followed by lysis in 1% NP40 detergent. Lysates were resolved by SDS-PAGE under non-reducing and reducing conditions. Immunoblotting with the anti-V5 antibody revealed HLA-B*27:05.HC and the HLA-B*27:05.SCT were capable of forming high molecular weight complexes, which were not apparent following reduction (**Figure 2A**).

To determine the composition of these dimeric structures, we examined lysates generated as above, from HeLa.HLA-B*27:05.HC and SCT expressing cell lines using non-reducing two-dimensional isoelectric focusing gel electrophoresis, followed by immunoblotting for HLA-B27. HC-dimers should retain an identical isoelectric point (pI) to monomeric heavy chain, whereas a heavy chain complexed to another protein would alter its overall pI. Immunoblotting revealed that the HLA-B27 signal resolved as monomers and dimers with similar pI, thus supporting that the high molecular weight complexes detected in both HLA-B*27:05 HC and SCT expressing cells were indeed HC-dimers. Additionally, we also identified that not every HLA-B27 monomer species seems capable of forming a dimer (**Figure 2B**).

To determine whether these dimers were ER resident, lysates were digested with endoglycosidase H (endo H) which cleaves ER associated but not post-ER sugar modifications. Endo H digested lysates were then resolved by non-reducing SDS-PAGE. Following digestion with endo H, dimers exhibited an apparent lower Mw than the corresponding undigested complexes, indicating that the majority of both HLA-B*27:05.HC and HLA-B*27:05.SCT dimers were indeed ER resident (**Figure 2C** **, arrows i-iii and iv-v**). To further assess the level of endo H resistant material at steady state with respect to total HLA-B27.HC and SCT molecules, we performed PNGase F and endo H digests which were subsequently analysed under both reducing and non-reducing conditions. PNGase F would remove almost all N-linked glycans irrespective of cellular location. A comparison of glycoproteins digested with endo H and PNGase F would provide an indication of the proportion of molecules with a mature phenotype with respect to their glycosylation status. Therefore, immunoblotting analysis revealed that a large proportion of HLA-B*27:05.HC and SCT molecules remained endo H susceptible at steady state (**Figures 2D, E** **)**. We also compared PNGase F and endo H digests of HLA-B*27:05.SCT to SCT molecules exhibiting efficient folding (HLA-B*35:01 and HLA-B27.F pocket). In comparison to efficient folding SCT molecules, immunoblotting revealed a significant proportion of endo H susceptible HLA-B27.SCT molecules (**Supplementary Figure 1**). Furthermore, PNGase F digestion resulted in a small reduction in Mw of each high Mw complex, indicating that these molecules did not represent glycan isoforms of the same complex (**Figures 2D, E** **)**.

To assess the possible expression of HLA-B*27:05.SCT dimers at the plasma membrane, we cell-surface biotinylated HeLa.E84, HeLa.HLA-B*27:05.HC and HeLa.HLA-B*27:05.SCT cell lines, followed by 1% NP40 lysis and immunoprecipitation with the conformationally specific antibodies W6/32 and ME1 and with HC10, which detects partially unfolded/folded HLA-B and -C alleles (61). Immunoprecipitates were then resolved by non-reducing SDS-PAGE and probed with streptavidin-Dylight 680 to detect cell surface biotinylated proteins. Immunoblotting did not reveal any cell surface expression of dimeric complexes which were W6/32, ME1 and HC10 reactive consistent with the endo H digestion analysis (**Figure 2F**).

### HLA-B*27:05 Expressed as a Single Heavy Chain And as a Single Trimeric Chain Forms Dimeric Complexes Within The Peptide Loading Complex

**Figure 3:**
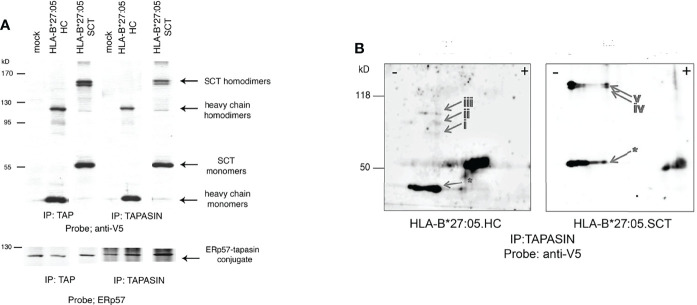
HLA-B*27:05 monomeric and dimeric complexes can associate with ER resident chaperones and within the PLC. **(A)** TAP and tapasin immunoprecipitations immunoblotted for HLA-B*27:05.HC and HLA-B*27:05.SCT reveal that both dimers and monomers can be detected within the PLC. HLA-B*27:05.SCT and HLA-B*27:05.HC dimers and monomers are indicated with arrows. TAP and tapasin immunoprecipitates were probed with ERp57, highlighting the ERp57-tapasin conjugate. **(B)** Two-dimensional isoelectric focusing gel analysis of tapasin immunoprecipitates immunoblotted with anti-V5 antibody reveal that both HLA-B*27:05 HC (arrows i-iii) and SCT (arrows iv-v) form homodimers within the PLC. Respective HLA-B*27:05.HC and SCT monomers are indicated (arrow *).

Few, if any studies have examined chaperone interactions with SCT MHC class I molecules during their assembly. We therefore examined the formation of the PLC, a key stage in optimal cell surface expression of MHC class I molecules. Thus, HeLa cell lines expressing HLA-B*27:05 HC or SCT were pre-treated with NEM, before lysis in the non-ionic detergent digitonin to help preserve the PLC. The PLC was subsequently immunoprecipitated with either anti-TAP or -tapasin antibodies. Immunoblotting for HC revealed HLA-B*27:05.SCT does indeed associate with the PLC. Surprisingly, we also detected HLA-B*27:05.SCT and HC high molecular weight complexes within the PLC (**Figure 3A**). We then performed tapasin immunoprecipitations and non-reducing two dimensional isoelectric focusing gel electrophoresis analysis. We reasoned that if these HC-dimers were composed of proteins other than MHC class I molecules, the monomeric form of both HLA-B*27:05.HC and SCT would run in the absence of a correlating dimer band. Following immunoblotting with the anti-V5 antibody, we detected dimeric conformers correlating to homodimers rather than mixed protein dimers (**Figure 3B**). Therefore HLA-B*27:05 can dimerise when expressed as an SCT during the latter stages of folding. This, to our knowledge is the first report of detection of dimeric MHC class I heavy chain structures in association with the PLC.

### Unpaired Cysteines Do Not Contribute to HLA-B*27:05.SCT Dimerisation

**Figure 4:**
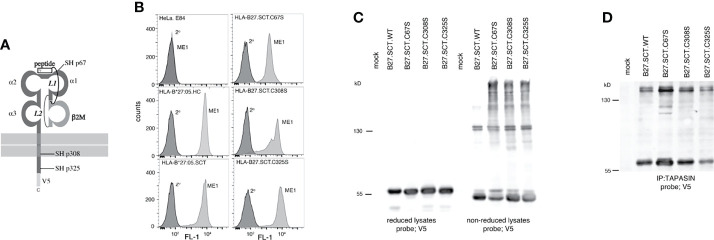
Unpaired cysteines at p67, p308 and p325 do not alter the ability of the HLA-B27.SCT to dimerise within the PLC. **(A)** Diagram illustrating the unpaired cysteine (-SH) residues at p67, 308 and 325 within the HLA-B27.SCT molecule. **(B)** Flow cytometric analysis of HeLa cells expressing HLA-B*27:05 heavy chain, HLA-B*27:05.SCT, B27.C67S.SCT, B27.C308S.SCT and B27.C325S.SCT. All constructs were detected by staining with the conformationally dependent ME1 antibody. Note HeLa cells were negative for ME1 staining. **(C)** Mutating each of the unpaired cysteines does not alter the dimerisation of the HLA-B27.SCT as detected in whole cell lysates. Immunoblotting of NEM treated cell lysates with the anti-V5 antibody revealed high molecular weight dimeric structures for HLA-B*27:05.SCT and the respective cysteine mutants. Dimeric structures are not evident following reduction. **(D)** HLA-B27.SCT cysteine mutants dimerised within the PLC. HeLa cells expressing the respective HLA-B27.SCT cysteine mutant constructs were NEM treated followed by lysis in 1% digitonin. Each was immunoprecipitated with anti-tapasin antibody, resolved by non-reducing 8% SDS-PAGE and immunoblotted with the anti-V5 antibody.

HLA-B27 expresses unpaired cysteine residues at p67, p308 and p325 which could drive SCT dimerisation (**Figure 4A**). We mutated these cysteines to serine residues, generated HeLa cell lines expressing two copies of the HLA-B27.SCT.C67S, B27.SCT.C308S and B27.SCT.C325S constructs and analysed their effects on dimerisation. Each of these mutant SCTs was expressed at the cell surface, with HLA-B27.SCT.C308S exhibiting some change in expression (**Figure 4B**). Each cell line was then treated with NEM prior to lysis in 1% NP40 detergent, resolved by non-reducing and reducing SDS-PAGE and immunoblotted for MHC class I HC with the anti-V5 antibody. Immunoblotting revealed all three cysteine mutant SCT molecules maintained their ability to dimerise. Following reduction, HC-dimers were disrupted and undetectable (**Figure 4C**). To assess whether HLA-B27.SCT.C67S, HLA-B27.SCT.C308S or HLA-B27.SCT.C325S participated in dimerisation within the PLC, tapasin was immunoprecipitated as already described, followed by immunoblotting for the respective SCT molecules with the anti-V5 antibody. Immunoblotting revealed all three SCT molecules could dimerise within the PLC (**Figure 4D**).

### Residues 114-116 of the HLA-B27 F Pocket Modulate Single Chain Trimer Dimerisation

**Figure 5:**
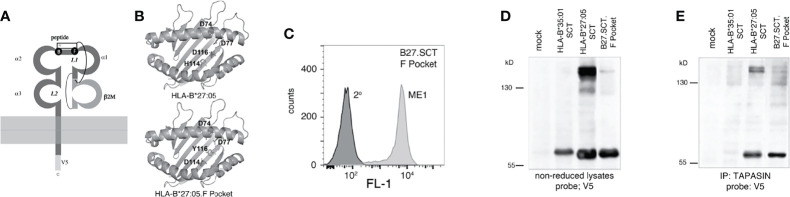
F pocket residue substitutions of H114D and D116Y alter the ability of the HLA-B27.SCT to dimerise within the PLC. **(A)** Diagram illustrating the B and F pockets within the HLA-B27 SCT. **(B)** Ribbon structure of HLA-B27 antigen binding groove generated from pdb file 5ib2 for HLA-B*27:05 illustrating the following residues (stick) H114 and D116 and additional D74 and D77 residues contributing to the overall charge of the F pocket (top panel). Similar structure illustrating D114 and Y116 residues of the HLA-B27.F pocket SCT along with the aforementioned aspartic acid residues. **(C)** Flow cytometric analysis of HeLa cells expressing HLA-B27.F pocket SCT as detected by staining with the conformationally dependent ME1 antibody. **(D, E)** F pocket substitutions alter HLA-B27.SCT dimerisation as detected in whole cell lysates and peptide loading complex. **(D)** Immunoblotting of NEM treated cell lysates with the anti-V5 antibody revealed high molecular weight dimeric structures for HLA-B*27:05.SCT but were diminished in the HLA-B27.F pocket SCT and were absent in the HLA-B*35:01 SCT. **(E)** HeLa cells expressing the respective SCT constructs were NEM treated and followed by lysis in 1% digitonin. Each was immunoprecipitated with anti-tapasin antibody. Immunoprecipitates were resolved by non-reducing 8% SDS-PAGE and immunoblotted with anti-V5 antibody.

The MHC class I SCT format allowed us to address whether changes within the HC could influence dimerization independently of the associated peptide. Previously, we demonstrated that in a peptide limiting system, F pocket residues greatly affected the ability of HLA-B27 to dimerise (48). The HLA-B27.SCT format allowed us to extend our previous observations and examine how F pocket changes affected HC folding in the presence of a single peptide. We therefore mutated p114 and p116 residues to aspartic acid (D) and tyrosine (Y) residues respectively. The D114 and Y116 changes correlated with the respective residues expressed by the HLA-B*27:06 subtype F pocket region (referred to as HLA-B27.F pocket SCT) **(** **Figures 5A** **, B** **)**. An isogenic HeLa cell line expressing two copies of HLA-B27.F pocket SCT was generated and flow cytometric analysis demonstrated HLA-B27.F pocket SCT expression at the cell surface **(** **Figure 5C** **)**. Lysates were generated from HLA-B*27:05.SCT, HLA-B27.F pocket SCT and HLA-B*35:01.SCT, resolved by non-reducing SDS-PAGE and immunoblotted with anti-V5 antibody. Immunoblotting revealed that dimerization of the HLA-B27.F pocket SCT was substantially reduced (**Figure 5D**). Immunoprecipitation of tapasin, followed by immunoblotting for V5 revealed HLA-B27.SCT.F pocket retained an association with the PLC, but with reduced detectable dimers compared to HLA-B*27:05.HC and HLA-B*27:05.SCT molecules (**Figure 5E**). Therefore, our data suggest that F pocket residues of pMHC.SCT molecules can influence their association with the antigen processing machinery.

### F Pocket Residues 114-116 of the HLA-B27 Single Chain Trimer Modulate Maturation Kinetics

**Figure 6:**
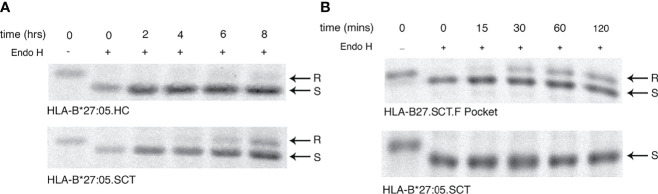
F pocket substitutions alter the maturation rate of HLA-B27.SCT. **(A)** Pulse chase analysis over 8 hrs followed by endo H digestion of anti-V5 immunoprecipitates demonstrate that HLA-B*27:05.HC has a slow maturation phenotype. HeLa cells expressing HLA-B*27:05.HC and HLA-B*27:05.SCT were metabolically labelled with ^35^S cys/met for 10mins, chased and immunoprecipitated with anti-V5 antibody for 0, 2, 4, 6 and 8 hrs, followed by digestion with endo H for 1hr at 37°C. Immunoprecipitates were resolved by reducing 8% SDS-PADE. Endo H sensitive (S) and resistant (R) conformers are indicated with arrows. The rate of endo H resistance acquisition by HLA-B*27:05.SCT does not significantly change until 8hrs of chase. **(B)** F pocket substitutions alter the maturation rate of HLA-B27.SCT. Pulse chase analysis over 120 mins reveals that the F pocket substitutions lead to the acquisition of endo H resistant (R) conformers after 30 minutes.

Rapid maturation kinetics have been correlated with reduced HLA HC-dimerisation (3). Therefore we examined the effects of p114 and p116 on the maturation kinetics of HLA-B*27:05.SCT, by performing pulse-chase analysis followed by endo H digestion. Initially, HeLa.HLA-B*27:05.HC and -B*27:05.SCT were metabolically labelled, chased for 0, 2, 4, 6 and 8 hours, immunoprecipitated with the anti-V5 antibody and digested with endo H prior to resolution by reducing SDS-PAGE. Surprisingly, our analysis revealed that HLA-B*27:05.SCT did not mature significantly faster than HLA-B*27:05.HC (**Figure 6A**). Next, we determined the effect F pocket changes had on SCT maturation. Metabolically labelled cells were immunoprecipitated with the anti-V5 antibody at 0, 15, 30, 60 and 120 mins, endo H digested and resolved by reducing SDS-PAGE. The HLA-B27.F pocket SCT molecule acquired endo H resistant conformers by 30 mins, despite associating with the same NP peptide as HLA-B*27:05.SCT (**Figure 6B**). These observations demonstrated that F pocket residues could profoundly affect the folding ability of MHC class I molecules in the SCT format. Collectively, these observations suggest that the ability of HLA-B27 to fold and propensity to dimerise is an intrinsic property of the HC.

## Discussion

Our study employed SCT molecules expressed using a controlled transfection system (**Figure 1**) and under our experimental conditions, demonstrated key features similar to previously designed SCT molecules (**Figure 1**) (17, 25, 58, 59). As SCTs represent preassembled MHC class I-peptide complexes, they have been proposed to bypass antigen processing and cellular quality control mechanisms prior to cell surface expression (23, 28). Synthesis of SCT molecules would require peptide synthesis prior to both β2m and HC and represent a single larger Mw protein requiring the intricate folding processes to attain an MHC class I-β2m-peptide complex. We envisaged that such a protein could potentially require chaperone assistance, however, little information exists regarding SCT folding. SCT molecules have clinical translational applications in the form of vaccines and immunotherapeutic interventions (23, 28, 30, 31, 59, 62–64), therefore, it is crucial to assess whether these molecules are truly independent of ER quality control mechanisms. As HLA-B27 is an MHC class I HC which can misfold (3, 45), by analysing various HLA-B27 SCT molecules, we were able to assess whether SCT molecules could overcome potential HC folding problems and simultaneously establish their folding requirements. In addition, HLA-B27 misfolding has been implicated in SpA development (34, 35), therefore we exploited the SCT format to express HLA-B27 and address the intrinsic folding/misfolding capabilities of the HC in a system where the peptide was fixed. Our observations demonstrated that MHC class I molecules in the SCT format can vary in their association with the intracellular antigen processing machinery. HLA-B27.SCTs demonstrated an ability to form dimer complexes and exhibit slow maturation rates, which were primarily driven by the HC rather than peptide. Moreover, HC-dimers could be detected at the latter stages of HLA-B27 assembly.

Our study is the first demonstration that dimers of HLA-B*27:05.HC or in the SCT format can be detected within the PLC, with F pocket residues influencing PLC associations and maturation kinetics. Whether these dimeric complexes detected within the PLC represent molecules with a folded or unfolded phenotype remains to be determined. Previously, HLA-B27 was demonstrated to form both ‘unfolded’ and ‘folded’ dimers as detected by HC10 and W6/32 antibodies respectively. HC10 and W6/32 antibodies can detect partially folded/unfolded and fully folded molecules respectively. Furthermore, HC10 reactive populations tended to accumulate in the absence of tapasin and β2m, whist the W6/32 ‘folded’ dimers depended on the presence of TAP, tapasin and β2m (39). Our observations with respect to both HC and SCT-dimers could therefore reflect these different HC-dimeric conformers and the complex nature of HLA-B27 dimerisation (65). As we employed an unbiased approach with respect to the detection of HLA-B27 conformations, the HC-dimers detected in cell lysates could reflect both ‘unfolded’ and ‘folded’ dimers (**Figure 2**). The dimeric HC and SCT populations detected within the PLC could well be the ‘folded’ conformations, with our study placing a pool of these molecules within the PLC. It is possible that only MHC class I molecules that have attained a more folded phenotype can enter and/or associate with the PLC. This would also explain the detection of more homogenous populations of monomeric HC detected within the PLC (**Figure 3**). However, we cannot discount that ‘folded’ dimers may form within the PLC due to the activity of the oxidoreductase ERp57 and PDI which form part of this complex (4, 7, 66). It was intriguing to note that not all HLA-B27 HC and SCT monomers could form high Mw complexes. The various HC monomers detectable by two-dimensional isoelectric focusing gel electrophoresis probably reflect post-translational modifications such as heterogenous sialylation. It was possible that other protein(s) do indeed interact with HLA-B27 and do not grossly affect the pI due to instability in the SDS- PAGE second dimension.

The glycosylation analysis of HLA-B*27:05 SCT did reveal that a substantial proportion of these molecules remain within the ER at steady state, much like the native heavy chain. Furthermore, the glycosylation status revealed the high molecular weight complexes were probably retained within the ER. The differences in molecular weight of the HC-dimers were not accounted for by different glycan isoforms. These observations agree with previous observations, where variations in redox status and/or folding could account for the different apparent molecular weights observed for HLA-B27 HC-dimers (43, 44, 46).

Mutagenesis analysis revealed each unpaired cysteine did not alter dimerization (**Figure 4**), consistent with earlier observations, where HLA-B27 HCs lacking C67, C308 and C325 retained the ability to dimerise (43, 44). These observations are consistent with previous findings regarding ER resident dimers, with the structurally conserved cysteines within the α2 domain, especially C164, having a significant role in HC-dimer formation (3, 44). The involvement of C164 and possibly C101 could reflect the disordered and flexible nature of the HLA-B27 antigen binding groove (67–70), as well as the C101-C164 bond forming at a later stage during the folding process and remaining partial oxidized/reduced within the PLC (9, 10). Identifying HC-dimeric structures within the PLC suggested that these conformations could transit to the cell surface. However, our cell surface biotinylation and endo H digestion analysis taken together with previous observations, indicate that HC-dimers do not transit from the ER to the cell surface (3). Our analysis does not discount HC-dimers from forming post transit to the plasma membrane and/or following recycling *via* endosomal compartments (47). The apparent lack of cell surface HC-dimers could be due to the loss of antibody epitopes as a result of recycling through acidic compartments or in the case of W6/32, biotinylation could partially disrupt the antibody epitope (39). However, we have previously demonstrated that biotinylated cell surface HC-dimers can be immunoprecipitated by HC10 antibody (3).

Our observations with HLA-B27.SCT interactions within the PLC were somewhat surprising. With regard to the tapasin dependence of HLA-B27 and its subtypes, conflicting reports exist (15, 70–72). Tapasin dependency has been based on MHC class I expression in the presence or absence of this MHC class I specific accessory molecule. However, being tapasin independent would not necessarily mean that such MHC class I molecules would not interact with the accessory molecule when it is expressed. HLA-B*27:05 was co-immunoprecipitated with TAP (71, 72), suggesting an interaction with tapasin. Indeed, when tapasin is available, HLA-B*27:05 demonstrates increased thermal stability, which demonstrates that HLA-B*27:05 profits from the interaction (15, 70), whilst expression of HLA-B*27:06 F pocket residues were proposed to reduce tapasin dependency (72). Recent evidence suggests dependence on tapasin is governed by, conformational instability in MHC class I molecules in a peptide-free state, F-pocket acidity and subsequent recognition of a conserved allosteric site at the base of the α2 domain which allows for peptide exchange (70, 73, 74). It is possible HLA-B27 could fall into a category of MHC class I alleles that could be partially tapasin dependent/independent. The interactions of HLA-B27.SCT molecules with the PLC could be a reflection of the slow maturation rate which may result in a slow on and off rate from the PLC. Tapasin also reduces the conformational disorder of empty or sub-optimally loaded MHC class I molecules, mediating iterative peptide exchange by accelerating peptide dissociation (70, 75, 76). The antigen binding site of HLA-B*27:05 is conformationally disordered in the absence of peptide due to a charge repulsion at the base of the F pocket (70). We detected both HLA-B*27:05 and the F pocket SCT molecules within the PLC, following co-immunoprecipitation of tapasin (**Figure 4**). Whether such conformational disorder remains in the HLA-B*27:05.SCT is undetermined but may explain the associations with the PLC. Our observations are somewhat unexpected, considering SCT molecules should theoretically assemble with peptide without the need for the PLC. These observations could be explained if there was some intrinsic feature of MHC class I molecules necessitating or directing PLC associations.

Changes to residues proposed to reduce tapasin requirements and alter the conformational dynamics of the binding groove did not abrogate HLA-B27 PLC associations and such characteristics may not be unique to HLA-B27. Other MHC class I molecules such as HLA-B*44:05, which have also been described as relatively tapasin-independent (77), can co-precipitate with the PLC (78). However, we cannot exclude that these residue changes could improve the stability of the peptide-binding groove without altering the interaction between HC and the PLC. Residues 74, 77 and 116 contribute to the electrostatic potential of the F pocket. In HLA-B*27:05 p74, p77 and p116 are all aspartic acid residues, resulting in a strong negatively charged F pocket (**Figure 5A** **, B** **. top panel**). In the HLA-B27.F pocket SCT, Y116 results in the negative potential being concentrated within the α1 helix region of the pocket (**Figure 5B** **, bottom panel**). The concentration of the negative potential in the α1 helix region could possibly result in a less disordered α2-1 helix region, which tends to be stabilized by tapasin binding and required for peptide optimisation by both tapasin and TAPBPR (12, 76, 79). The impact of such changes in HC residues could influence cell mediated immunity. For example, an HLA-A2 SCT expressing a histidine (H) to leucine (L) change at p74 (H74L) presenting a tumour associated peptide, led to enhanced *in vivo* priming and protection against tumour challenge (24). Though SCTs have been engineered to stabilize peptide by introducing artificial disulfide traps (23, 28, 80, 81), our study along with those of Matsui and colleagues, raises the possibility of employing alternative changes to the HC to alter immunogenicity and stability.

F pocket mutations were able to abrogate HC-dimer formation of the HLA-B27.SCT, suggesting that mechanisms other than changing the peptide repertoire could influence HC-dimer formation. Studies employing the HLA-B*44:02 and 05 subtypes proposed that p116 residues strongly influenced the flexibility of the antigen binding cleft (19, 76). Moreover, HLA-B*27:05 in comparison with HLA-B*27:09, revealed enhanced dynamic flexibility of the α-helices surrounding the peptide-binding groove (70). Our observations would support a previous proposal that differential dynamic behaviour of the HLA–B27 subtypes at physiological temperature was accounted for by intrinsic HC attributes and not by peptide sequences (67).

Alternatively, the F pocket mutations could alter the affinity between NP_383-391_ and the antigen binding groove. P116 was shown to be important for the interaction of NP_383-391_ with HLA-B27 and an aspartic acid to phenylalanine point mutation decreased the stability and affinity of the HLA-B27-NP peptide complex. The observed instability was driven by the loss of an acidic charge within the F pocket, leading to a decrease in the allowance for R9 polar residues (82). The D116Y mutation could alter the affinity for HLA-B27.SCT binding the NP peptide, however, the HLA-B*27:06 changes also included an acidic D114 residue. The rationale of using the SRYWAIRTR peptide was that it exhibits high binding kinetics/affinities to HLA-B*27:05 which are not significantly different to other HLA-B27 subtypes including HLA-B*27:06 and are within the range of most HLA class I binding peptides (57). Though the EC_50_ of SRYWAIRTR peptide binding to HLA-B*27:05 and 06, was not significantly different, the HLA-B*27:06 subtype exhibited a reduced affinity for this influenza derived peptide. However, when comparing SRYWAIRTR binding to HLA-B*27:04 and 06, which would be equivalent to comparing the impact of the D114 and Y116 residues of the F pocket, there were few, if any differences in the EC_50_ values (57). Therefore, it is possible that SRYWAIRTR binding affinities are not significantly different between the HLA-B*27:05 and 06 F pocket SCT. However, HLA-B27 peptide affinities could influence whether HLA-B27 is predisposed to dimerisation. Therefore, it is intriguing to speculate that the ERAP1 associations with inflammatory arthritis may not govern the presentation of arthritogenic peptide(s) postulated to stimulate autoimmune response(s), but instead, influence the production of peptides that may exacerbate or instigate HLA-B27 misfolding.

A surprising feature of the HLA-B27 SCTs was the notable differences in maturation rates. SCTs have been demonstrated to fold rapidly (17). In the case of HLA-B27, though the SCT does mature to some extent more quickly than the B27.HC, the D114Y116 changes dramatically impact on the maturation of the HLA-B27 SCT molecule (**Figure 6**). These findings are consistent with our previous observations made in an HLA-B27 peptide limiting system (48). Furthermore, the enhanced maturation rates that we have observed, are consistent with findings suggesting the HLA-B*27:06 subtype matures more rapidly than disease associated subtypes such as HLA-B*27:05 (72, 83). It must be noted we have employed a controlled expression system and an unbiased approach in capturing most, if not all populations of HLA-B27, as we employed an antibody directed to the C-terminal tag which is not dependent on specific subpopulations of HLA-B27 (84). Taken together, the rapid acquisition of endo H resistance by the HLA-B27.F pocket SCT and a lack of HC-dimerization, further supports the hypothesis that MHC class I HC maturation rates can determine their ability to form HC aggregates (3).

Our observations suggest that there is an intrinsic feature within HLA-B*27:05.HC which can, under the appropriate conditions, lead to enhanced misfolding. HLA-B27 expressing rats, when provided with the SRYWAIRTR expressed as a transgene, led to reduced prevalence of arthritic disease with no effect on gastrointestinal inflammation (85). When folding was improved in these rat models by providing additional human β2m, there was an increase in prevalence and severity in arthritis with no effect on gastrointestinal inflammation. Intriguingly, a healthy HLA-B27 rat line expressing less transgene copies, when provided with extra human β2m exhibited a severe arthritis but no colitis. Such observations suggest HLA-B27 misfolding may have a predominant role in intestinal epithelial cellular inflammatory responses (86). Our studies could explain why, even in the presence of a high affinity HLA-B27 binding peptide, gastrointestinal symptoms were not alleviated in HLA-B27 transgenic rats and that these clinical features may be more associated with HLA-B27 misfolding.

## Data Availability Statement

The original contributions presented in the study are included in the article/**Supplementary Material**. Further inquiries can be directed to the corresponding author.

## Author Contributions

IL designed and performed experiments and contributed to the writing of the manuscript. L-HT designed and performed experiments and contributed to the writing of the manuscript. DN designed and performed experiments and contributed to the writing of the manuscript. OR performed experiments and contributed to the writing of the manuscript. ET designed experiments and generated reagents.

JA performed experiments. PK performed experiments. KM performed experiments and contributed to the writing of the manuscript. BP performed experiments. BM designed experiments and contributed to the writing of the manuscript. MG performed experiments and contributed to the revision of the manuscript. PB generated reagents, designed experiments and contributed to the writing of the manuscript. NB performed experiments and contributed to the writing of the manuscript. SP designed and performed experiments and contributed to the writing and the revision of the manuscript. KG designed experiments and contributed to the writing of the manuscript. DN designed experiments, generated reagents and contributed to the writing of the manuscript. AA designed and directed experiments and wrote and revised the manuscript. All authors contributed to the article and approved the submitted version.

## Funding

IL was supported by Versus Arthritis studentship (17868), AA was supported by an Versus Arthritis Fellowship (15293). PK was supported by Breast Cancer Now UK (2018JulPR1086).

## Conflict of Interest

The authors declare that the research was conducted in the absence of any commercial or financial relationships that could be construed as a potential conflict of interest.

## Publisher’s Note

All claims expressed in this article are solely those of the authors and do not necessarily represent those of their affiliated organizations, or those of the publisher, the editors and the reviewers. Any product that may be evaluated in this article, or claim that may be made by its manufacturer, is not guaranteed or endorsed by the publisher.

## Supplementary Material

Supplementary Figure 1 HLA-B*27:05 SCT exhibits a large proportion of endo H susceptible molecules compared to HLA-B*35:01 and HLA-B27.F pocket SCT molecules. Lysates digested with PNGase F and endo H were resolved by reducing SDS-PAGE, followed by immunoblotting with anti-V5 pK antibody. Immunoblotting revealed that a large proportion of HLA-B*27:05 SCT molecules were susceptible to endo H digestion at steady state compared to similarly treated HLA-B*35:01 and HLA-B27.F pocket SCT molecules.
