# Supplementary figures and images for: Intrinsic Folding Properties of the HLA-B27 Heavy Chain Revealed by Single Chain Trimer Versions of Peptide-Loaded Class I Major Histocompatibility Complex Molecules
Source: Front Immunol. 2022 Jul 25;13:902135. doi: 10.3389/fimmu.2022.902135 (PMC9359109; doi:10.3389/fimmu.2022.902135)

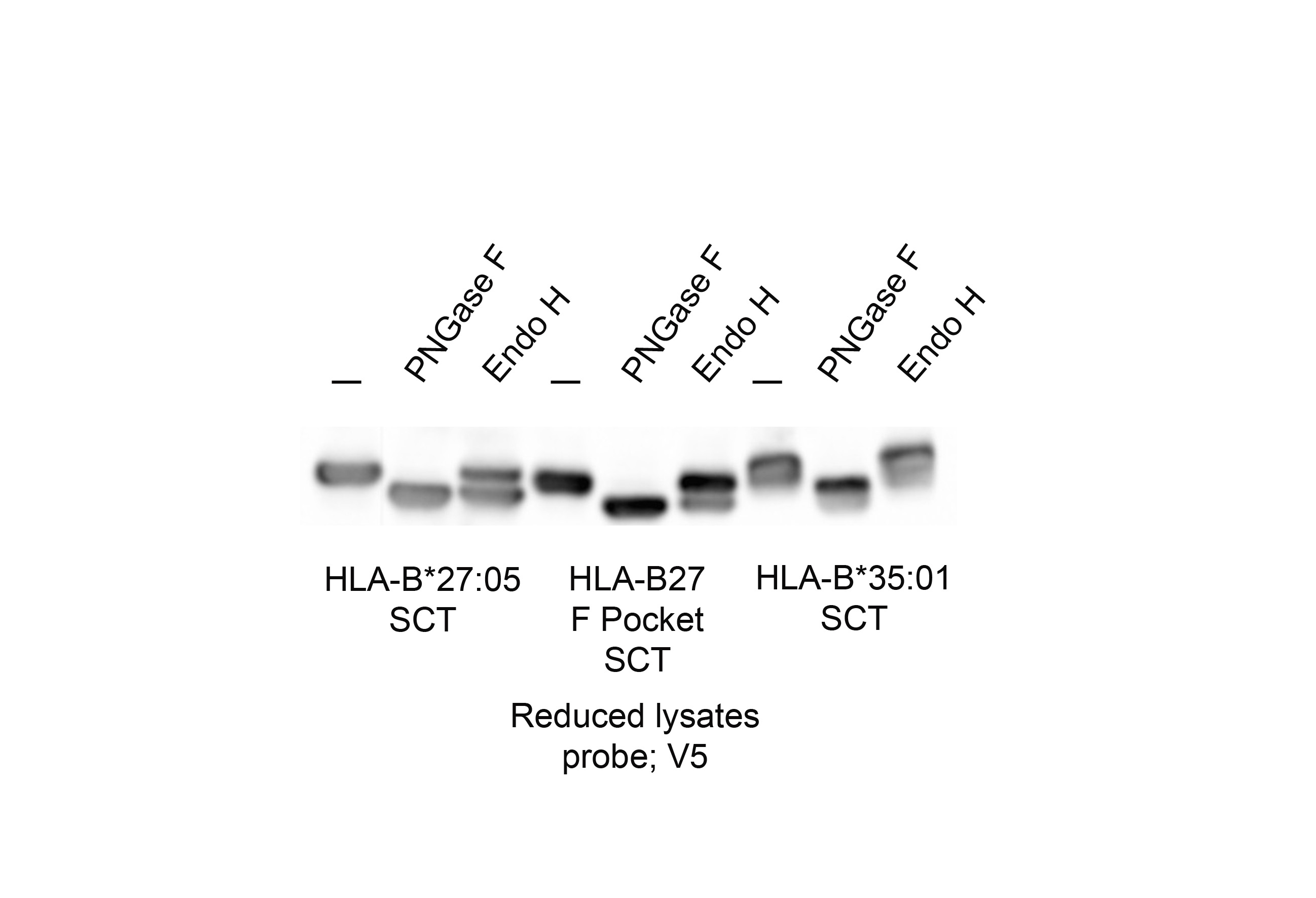

Supplement: Supplementary Figure 1 — HLA-B*27:05 SCT exhibits a large proportion of endo H susceptible molecules compared to HLA-B*35:01 and HLA-B27.F pocket SCT molecules. Lysates digested with PNGase F and endo H were resolved by reducing SDS-PAGE, followed by immunoblotting with anti-V5 pK antibody. Immunoblotting revealed that a large proportion of HLA-B*27:05 SCT molecules were susceptible to endo H digestion at steady state compared to similarly treated HLA-B*35:01 and HLA-B27.F pocket SCT molecules. [file Image_1.jpeg]
